# Automatic medical specialty classification based on patients’ description of their symptoms

**DOI:** 10.1186/s12911-023-02105-7

**Published:** 2023-01-20

**Authors:** Chao Mao, Quanjing Zhu, Rong Chen, Weifeng Su

**Affiliations:** 1 Guangdong Provincial Key Laboratory of Interdisciplinary Research and Application for Data Science, BNU-HKBU United International College, Zhuhai, 519087 China; 2 Specialty of Laboratory Medicine, West China Hospital, Sichuan University, Guoxue Lane, Wuhou District, Chengdu, 610041 China; 3 Specialty of Rehabilitation Medicine, The First Affiliated Hospital, Sun Yat-sen University, Guangzhou, 510080 China

**Keywords:** Medical specialty classification, Convolutional neural network, BERT, Attention, Registration

## Abstract

In China, patients usually determine their medical specialty before they register the corresponding specialists in the hospitals. This process usually requires a lot of medical knowledge for the patients. As a result, many patients do not register the correct specialty for the first time if they do not receive help from the hospitals. In this study, we try to automatically direct the patients to the appropriate specialty based on the symptoms they described. As far as we know, this is the first study to solve the problem. We propose a neural network-based model based on a hybrid model integrated with an attention mechanism. To prove the actual effect of this hybrid model, we utilized a data set of more than 40,000 items, including eight departments, such as Otorhinolaryngology, Pediatrics, and other common departments. The experiment results show that the hybrid model achieves more than 93.5% accuracy and has a high generalization capacity, which is superior to traditional classification models.

**Supplementary Information:**

The online version contains supplementary material available at 10.1186/s12911-023-02105-7.

## Introduction

In China, there are very few general practitioners available for the patients. Most patients go to the hospital directly and register a corresponding specialist. In the process, the patients need to determine their specialty first, which usually entails a lot of medical knowledge. Consequently, many patients do not register the correct specialty and have to try to the correct specialty many times. In particular, many registrations are conducted online, which is unlikely to get help from the medical personnel. Lots of patients’ and doctors’ time and medical resources are wasted [1]. More importantly, the patient’s life chances may be missed. Therefore, it is significantly valuable to construct an automatic triage system to direct the patients to the correct specialty based on their symptoms [2]. The triage system can also reduce the workload and improve the efficiency of the medical staff in the hospital [3]. The system is also useful for the general practitioner and other medical personnel when it provides professional advice to aid decision-making.

We propose a Hybrid Model (HyM) for the classification of medical symptom descriptions. In HyM, the long short-term memory (LSTM), text convolutional neural networks (TEXT-CNN), bidirectional encoder representations from transformers (BERT) and term frequency–inverse document frequency (TF-IDF) were exploited to extract different features of patients’ symptom description. Thereafter, the features were combined to classify the patients to specialties according to the symptom description.

Our contributions can be highlighted as follows:

- This is the first study to automatic classifying the patients’ describing symptoms. The experimental results also show that the patients’ describing symptoms can be classified accurately and it can be very useful to effectively improve the work efficiency of the hospital.
- A novel model, HyM, is proposed. It is a hybrid model which effectively integrates the features extracted by LSTM, CNN, BERT and TF-IDF.
- A dataset is created which includes the Chinese symptom description utilized from offline hospitals and an online health service platform. Each description is professionally classified into one of eight medical specialties, such as Otorhinolaryngology, Pediatrics, and other common departments.

The rest of the paper is organized as follows. In section “Related works” summarizes related works on the topic of text classification and deep learning. In section “Models” describes the proposed model in details. In section “Experiment results and analysis” presents the utilized dataset, the experimental results and analysis. Finally, “Conclusion” section concludes the paper and provide some future works.

## Related works

Deep learning (DL) is widely used in the field of health with the development of artificial intelligence technology [4–6]. And text classification techniques play an increasingly important role in Cheng and Du et al. [7, 8] proposed a hybrid model to address a multi-label medical text classification, which improved the efficiency of text classification greatly. Actually, medical text data is complicated and filled with redundant information, making it difficult to extract relevant information from the massive volume of data. Chen [9] improved the ability to obtain key information from big data by incorporating attention mechanisms into medical text classification. However, there are still a lot of features utilized for categorization, which makes the process time-consuming and inaccurate. Mohasseb et al. [10] proposed a machine learning approach for question classification based on grammar. According to experimental findings, the suggested framework is effective in recognizing various question types and addressing the class imbalance. All in all, text categorization methods have advanced significantly in the field of medicine.

With the rise of online health services, the information asymmetry between doctors and patients and uneven geographical distribution of medical resources have become increasingly prominent [11]. Text categorization models can be created using internet data to increase the precision and efficacy of patients' medical decisions, and to enhance their medical experience. The first research of machine learning methods for text categorization using a primary care teleconsultation dataset is by López Segu et al. [12], which has significant theoretical and applied value. The study demonstrates how artificial intelligence-powered text analysis can be very beneficial for assisting medical practitioners with their decision-making. Data on interactions between patients and doctors are particularly valuable to improve the patient experience, Wei Lu et al. [13] proposed a model that combines patient feedback data with doctor activity data, which can help patients to choose appropriate department and doctors. This model provided a novel approach to data-driven telemedicine services. Hossam Faris [14] proposed a novel machine-learning approach based on question content to address the problems associated with medical multi-classification in QA systems. The test results indicate that the suggested approach dealt with difficult Arabic language issues incredibly successfully. However, online data produced by interactions between patients and doctors are underexplored in China.

According to the study of the relevant literature, there is a lack of research on the classification of symptom presentation within the Chinese medical specialty. However, the development of an automated triage system in China that routes patients to the appropriate specialty based on their symptoms is crucial and beneficial. Consequently, we proposed a project specific for this issue. After constructing the model, its accuracy and utility were evaluated using a local Chinese medical dataset including 40,000 items.

In order to assist readers in better comprehending this study, we included definitions of some common terms in DL before to the main text.

### Long short-term memory

The original LSTM paper appeared in 1997 [15]. It is a time-cyclic neural network, and LSTM was created specifically to address the generic recurrent neural networks's (RNN) long-term reliance issue (cyclic neural network) [16]. An input gate, an output gate, and a forget gate make up a typical LSTM cell [17]. Three gates regulate the flow of data into and out of the cell, which can retain values for any length of time. Because of its distinctive architectural structure, LSTM can process and forecast significant events in time series with very long intervals and delays.

In the field of natural language processing (NLP), people often use LSTM to extract the semantic and grammatical information of text, and then cooperate with downstream models to do specific tasks, such as classification, sequence labeling, and healthcare [18].

### Attention

Attention mechanism is based on the way humans think about attention and is widely used in the field of NLP. In 2014, the Google Mind team proposed to use the attention mechanism on the RNN model for image classification [19], and the results achieved very good performance. Bahdanau et al. proposed to use the attention mechanism to simultaneously perform translation and alignment on machine translation tasks [20]. Their work is the first to apply the attention mechanism to the NLP field. Since then, the attention mechanism has been widely used in various deep learning tasks based on the RNN neural network model. In 2017, Google proposed to use the self-attention mechanism extensively in machine translation to learn text representation [21].

### Bidirectional encoder representations from transformers

BERT is a transformer-based machine learning method for pre-training NLP [22]. BERT is capable of learning bidirectional representations of text, greatly enhancing its capacity to comprehend unlabeled text in context for a variety of applications. The 24 encoders and 16 bidirectional self-attention heads make up our standard BERT model.

### TF-IDF

TF-IDF is frequently used in text processing, to assess the significance of a term in a document. It is used to extract keywords from documents. A word's TF-IDF value is computed by multiplying its term frequency (TF) and inverse document frequency (IDF) values. By calculating the TF-IDF of each word in the article, sorting from large to small, and ranking in terms of importance, the larger the TF-IDF of a word in the article, the more essential the word will be in this article generally [23]. The article's keywords appear in the first few words. Frequently, this approach is utilized for text classification and recommender systems.

### TEXT-convolutional neural network

The Convolutional Neural Networks (CNN) is a subclass of feed-forward neural networks with a deep structure and convolution calculations [24]. In 2014, Yoon Kim made some deformations for the input layer of CNN and proposed a text classification model TEXT-CNN [25]. Compared with CNN, TEXT-CNN becomes simpler in network structure. The model is popular in the disciplines of text classification, recommender systems, and NLP due to its benefits of simplicity of design, sparse usage of parameters, and quick training times.

### Jieba

The process of breaking down written text into understandable components, such as words, sentences, or topics, is known as text segmentation [26]. When working with English text, it is possible to separate words directly by the spaces between words, as English text is inherently word-sorting. However, for Chinese and other languages with similar forms, we need to use tools to handle word segmentation [27]. Jieba library is an excellent Chinese word segmentation third-party library, and it is widely used in Chinese text classification tasks with good performance [28–30].

## Models

**Fig. 1:**
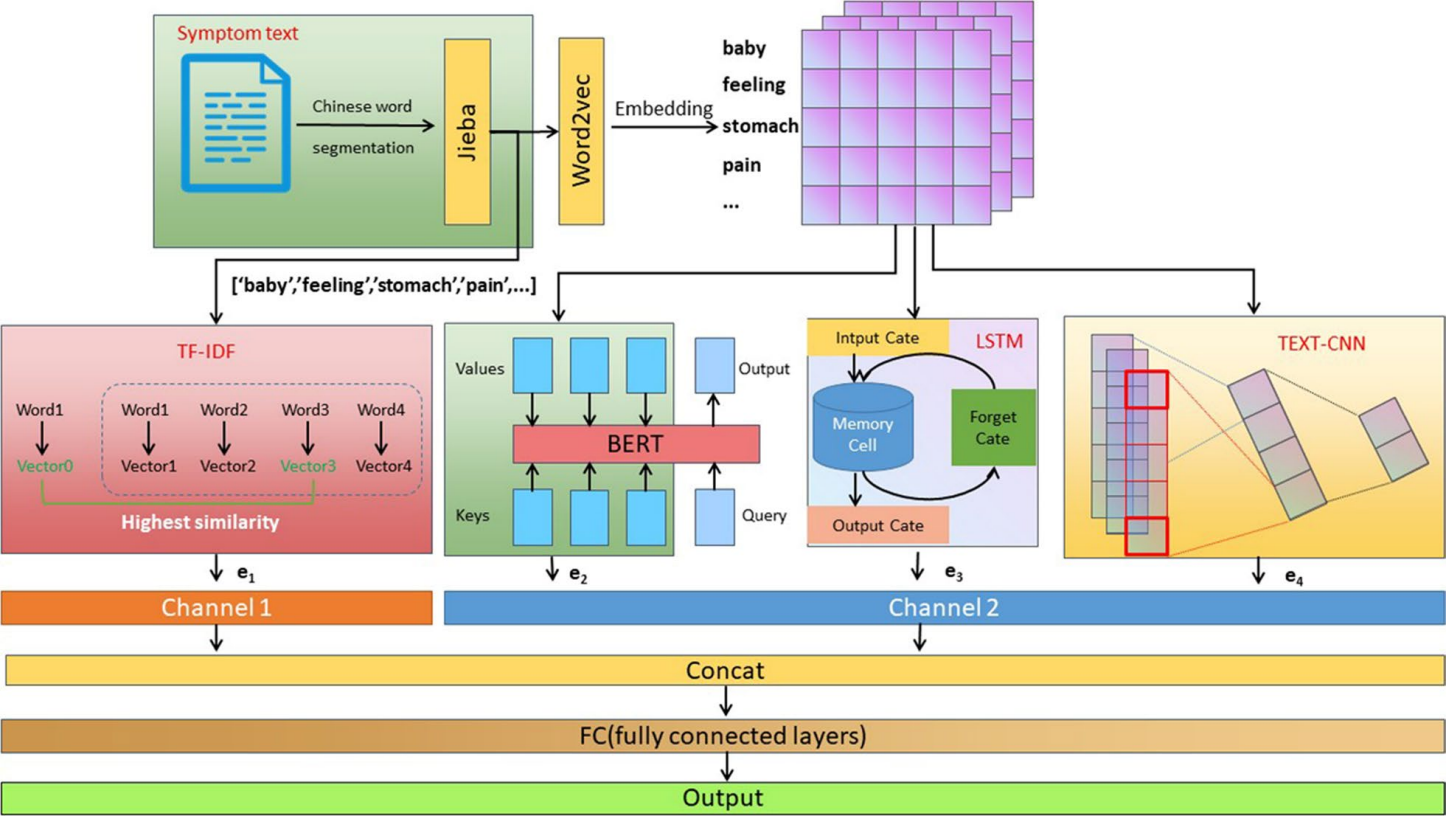
The overall architecture of the Hybrid methods, HyM, including TF-IDF, BERT, LSTM and TEXT-CNN, are applied to extract the features at different levels from the symptom texts. The features are concatenated and fed into a fully connected neural network for classification

The Fig. 1 shows the overall architecture of the proposed model, HyM. The first step is to extract the features for each symptom text. We hope that all the important information, such as context, semantic and syntax information, can be extracted for future processing. Hence, different methods are applied to extracted different levels of features. In particular, there are two major channels for feature extraction.

1. In the first channel, 10 features are generated using TF–IDF techniques without word vectorization. The TF-IDF methods can effectively capture the shallow level semantic information in the symptom texts.
2. In the second channel, the symptom texts are converted to vectors first. The vectors then are fed into three modules: BERT, LSTM and TEXT-CNN respectively. Each of the three modules generates 10 features. The second channels are good at capturing the deep level semantic information and context information in the symptom texts.

The features are concatenated to represent the symptom texts. A fully connected neural network is used as the classifier to classify the symptom text to the medical specialties.

### Text preprocessing and word vectorization

In the preprocessing step, the Jieba tool is used to segment the symptom description text into words. Thereafter, the punctuation marks, stop words, and special characters are removed.

To facilitate the future processing, the number of words for each symptom description text are all set to $L$. If a symptom description text has more than $L$ words, the words after $L$th are truncated. If a symptom description text has less than $L$ words, we pad the words with blank words so that the number of words is $L$. L is empirically set to be 50 in the experiments. After the preprocessing step, each symptom description text $s$ is represented as $L$ words ${w}_{1}, {w}_{2},\dots , {w}_{L}$.

The Word2Vec module creates a numerical value vector for each word ${w}_{i}$. The advantage of the vector representation is that words with similar characteristics are also in close proximity to one another as vector representation. This step is essential if we want to use the deep learning techniques to handle symptom description text. In the experiment, a dictionary is used and a word is converted to its corresponding vector by looking up its vector in the dictionary. That is, each word ${w}_{i}$ is converted into an N-dimensional numerical value vector ${V}_{i}$ and $s$ is represented as a matrix with size $*N$.

### Features extraction based on TF-IDF

The TF–IDF weighting technique has been widely used in the information retrieval and text mining areas. It is a statistical method for determining the importance of a word in a document. Its importance increases proportionally to the frequency of its appearance in the document but decreases proportionally to the number of documents it occurs in the corpus.

1 $$ {\text{TF - IDF}} = {\text{TF}} * {\text{IDF}} $$

After the word segmentation is done, the TF-IDF is used to extract the feature vector in the first channel (see Fig. 1). It also produces a vector with length $L$, which is 50 in the experiments. The details are illustrated in Additional file 1: Fig. S1. In this channel, the word's distinctive and significant position in the document is represented by the output vector characteristics.

### Features extraction based on TEXT-CNN

TEXT–CNN is a one-dimensional convolutional neural network specific for text processing. It has the same structure as an ordinary CNN. It has a convolutional layer, a pooling layer, and a fully connected layer in sequence. Text segmentation is used as the input for TEXT-CNN. The breadth of the filter is comparable to the width of the word matrix, which is created through embedding and contains vectors. The word vector's width determines its size, and the filter can only move in the height direction. The filter's breadth corresponds to the word vector's width. Finally, an external softmax will be created in order to achieve multi-classification. The model structure is displayed in Additional file 1: Fig. S2.

In this study, sentence vectors were trained using TEXT-CNN as the content expression layer, and convolutional features were chosen using maximum pooling with a stride of 1. The more significant elements of the text are retained, and some contextual information is preserved via a max-pooling with stride 1.

### Features extraction based on LSTM

**Fig. 2:**
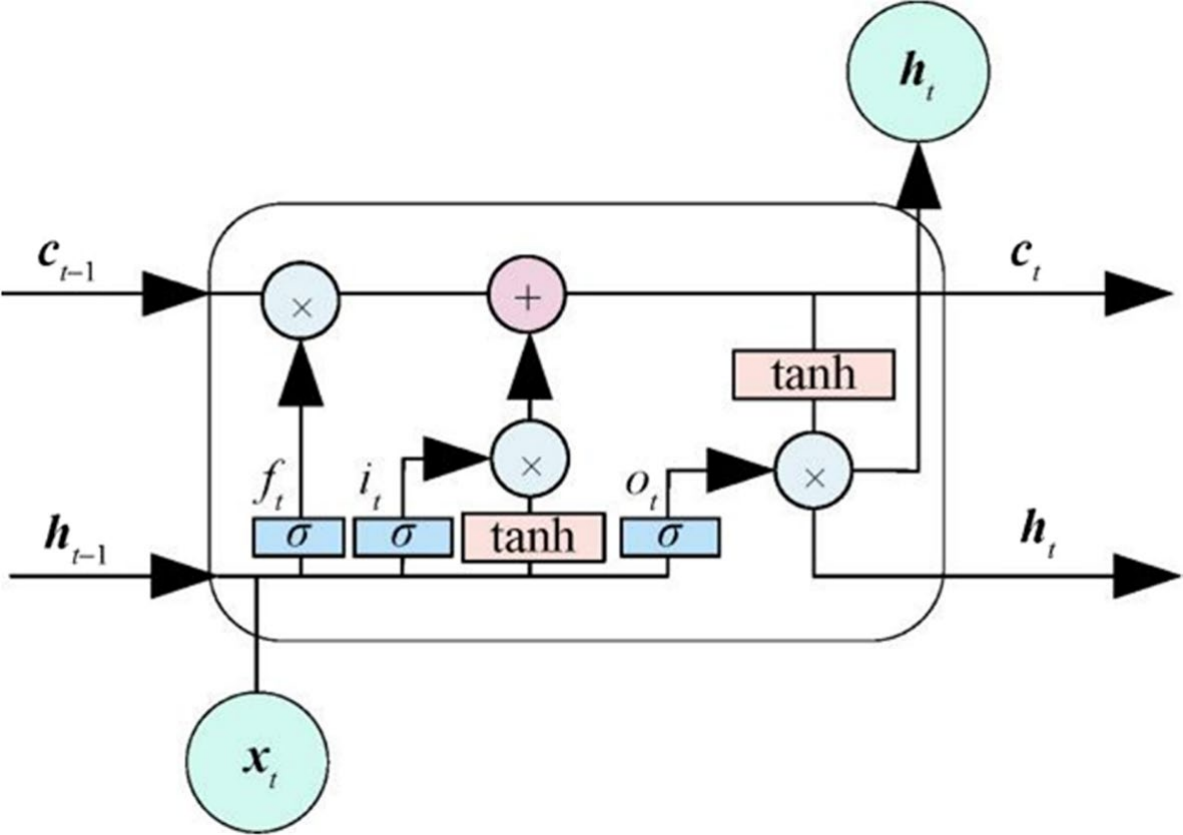
LSTM unit structure

RNN are good at processing sequence data. However, as the sequence length increases, it suffers from the problems of vanishing gradients, exploding gradients, and long-term dependencies and etc. As a variant RNN, as Fig. 2, LSTM addresses the aforementioned problems by adding the input gate i_t_, forgetting gate o_t_, output gate f_t_, and memory state cell c_t_ and uses the gate mechanism to control information retention, forgetting, and state update. The calculation formulas are listed as follows:

2 $$ {\text{f}}_{{\text{t}}} = \sigma \left( {{\text{W}}_{{\text{f}}} \cdot \left[ {{\text{h}}_{{{\text{t}} - 1}} ,{\text{x}}_{{\text{t}}} } \right] + {\text{b}}_{{\text{f}}} } \right) $$

3 $$ {\text{i}}_{{\text{t}}} = \sigma \left( {{\text{W}}_{{\text{i}}} \cdot \left[ {{\text{h}}_{{{\text{t}} - 1}} ,{\text{x}}_{{\text{t}}} } \right] + {\text{b}}_{{\text{i}}} } \right) $$

4 $$ {\text{o}}_{{\text{t}}} = \sigma \left( {{\text{W}}_{{\text{o}}} \cdot \left[ {{\text{h}}_{{{\text{t}} - 1}} ,{\text{x}}_{{\text{t}}} } \right] + {\text{b}}_{{\text{o}}} } \right) $$

5 $$ {\text{c}}_{{\text{t}}} = {\text{f}}_{{\text{t}}} \odot {\text{c}}_{{{\text{t}} - 1}} + {\text{i}}_{{\text{t}}} \odot \tanh \left( {{\text{W}}_{{\text{c}}} \cdot \left[ {{\text{h}}_{{{\text{t}} - 1}} ,{\text{x}}_{{\text{t}}} } \right] + {\text{b}}_{{\text{c}}} } \right) $$

6 $$ {\text{h}}_{{\text{t}}} = {\text{o}}_{{\text{t}}} \odot \tanh \left( {{\text{c}}_{{\text{t}}} } \right) $$

where σ is the nonlinear activation function, W is the weight matrix, b is the bias, x_t_ is the input vector at time t, h_t−1_ is the output at the previous time, c_t−1_ is the hidden state at the previous time, c_t_ and h_t_ are the current state and output, respectively.

LSTM effectively solves the long-term dependency problem of RNN and alleviates the ‘gradient disappearance’ problem caused by the backpropagation of RNN during training with the gates mentioned above.

LSTM is used in this study to represent the deep level features of disease symptom text as vectors (see Additional file 1: Fig. S3). LSTM encodes the sentence embedding input at each moment to obtain the corresponding hidden layer vector (the output dimension is (128, 10)) and obtains a new vector.

### Feature weighting based on BERT

The attention mechanism is designed to demonstrate the degree to which each feature word contributes to correctly categorizing the entire body of text into the intended category. The attention process gives priority to content that is vital, while ignoring content that is not as important as other content. The direct weighted average of the output vector and the addition of the attention mechanism to the network are contrasted; the former prevents the maintenance of redundancy, while the latter improves classification accuracy by retaining noise from the original text after the average has been taken into account. The Attention mechanism is utilized quite frequently within the BERT model. Within the context of this paper, the BERT model is used to compute the attention mechanism.

As illustrated in Additional file 1: Fig. S4, the BERT model was used to obtain attention-weighted vectors. This mechanism gives the output of the network model varied weights, which enhances the attention to keywords by incorporating word-level features further. The model's attention to local features and the expression of sentence semantic information can both be improved by the attention mechanism. In order to improve the accuracy of the final categorization, a word's weight will be increased if it contributes more meaning to the sentence; otherwise, its weight will be decreased.

A weighted semantic feature vector X, encompassing local and global features, is produced after the preceding model extracts the local and global feature representation of the text.

7 $$ {\text{X}} = {\text{Add}} < {\text{e}}_{2} ,\;{\text{e}}_{3} ,\;{\text{e}}_{4} > $$

where e_2_ stands for the feature vector produced by BERT e_3_ for the feature vector following the LSTM, e_4_ stands for the feature vector produced by TEXT–CNN.

### Model fusion and output

The above two-channel models extract the local and global feature representations of the symptom description text, which are then concatenated to produce the local and global feature vector, X.

After the two channels are fused, a fully connected layer, FC, is added to translate the weighted vector of symptom text features into the label space (Fig. 1). To avoid the weight update after the fully linked layer, a dropout mechanism is included that solely relies on partial features and model overfitting. We directly output the model prediction results after using a classifier to compute the probability distribution of the medical specialty classification to which the patient's symptoms belong.

## Experiment results and analysis

### Experimental dataset

A Chinese patient description text dataset was built in order to validate HyM, which aims to automatically direct patients to the correct specialty depending on the symptoms they described. The data we utilized for offline registration comes from two different websites. (1) Online database: the data of online patient consultation (www.39.net), on the online platform, the patients describe their symptoms in text, and then the professional staffs determine the specialties for them. We utilized a dataset of more than 30,000 items, including eight departments, such as the Department of Cardiovascular Diseases, the Respiratory Department, the Ear, Nose and Throat Department, Pediatrics, and other common departments. (2) Offline database: the data from offline hospital is posted online on the website (www.xywy.com), which originally from offline hospital. In the offline hospitals, patients will ask the triage nurses which specialty should be registered. And these data are then compiled by the hospital and uploaded to the website, which is fully open for patient reference. In this part, we utilized a data set of about 20,000 items, including information in eight hospital departments, such as the Department of Cardiovascular Diseases, the Respiratory Department, the Department of Otolaryngology, Pediatrics, and other common departments. Thereafter, each instance is double checked by the doctor from the specialty that it has been assigned to. If the specialty doctor has a different opinion, we will seek more professional advice. If there is still controversy, the instance is given up.

We choose eight most common registered specialties in the dataset, each of which has a sample instance in Additional file 1: Table S1. In total, 48,128 patient description texts and their medical specialties were analyzed in this study.

### Evaluation metrics

The evaluation indicators used are accuracy, precision, recall, F-score and confusion matrix. Accuracy is the ratio of the number of correct predictions to the total number of predictions. Precision is used to analyze the validity of the results. Recall is used to checks the completeness of the results, and the F-score is used to balances the precision and recall.

8 $$ {\text{P}} = {\text{TP}}/\left( {{\text{TP}} + {\text{FP}}} \right) $$

9 $$ {\text{R}} = {\text{TP}}/\left( {{\text{TP}} + {\text{FN}}} \right) $$

10 $$ {\text{F}} = 2 * {\text{PR}}/\left( {{\text{P}} + {\text{R}}} \right) $$

Among them, P is the precision rate; R is the recall rate; TP is the correct text prediction in terms of the number of correct classes; FP is the wrong text prediction in terms of the correct class number; FN is the correct text prediction in terms of the number of incorrect classes, and F-score is the harmony between the precision rate and the recall rate average values.

A confusion matrix is used to summarize the results of a classifier. For k-ary classification, it is a (k × k) table used to record the classification results of the classifier. Each row indicates the number of data instances belonging to this class. Each column of the confusion matrix indicates the number of data instances that has been predicted as this category. The number in cell (i, j) represents the number of instances which belong to class i while been classified to class j. A good classifier should have large numbers on cell (i, i) and small numbers on cells (i, j), i ≠ j. As there are 8 medical specialties in our investigation, the confusion matrix is a (8 × 8) matrix.

### Experiment analysis

For the purpose of evaluating the efficacy of the methodology used in this study, several classification model comparison experiments, including those using the TEXT-CNN, LSTM, and BERT models, are set up in this section. Compare the variations between the model used in this study and other models when classifying the symptoms of the patient.

The accuracy of each model experiment is shown in Additional file 1: Table S2. It is worth noting that HyM's accuracy, which reached 93.5%, which is significantly higher than that of other models. The neural network model TEXT-CNN, which is specifically used for text classification tasks, has 88.7% accuracy compared to the LSTM's 81.3%. With a 90.5% accuracy rate, the BERT model of the encoder employing the bidirectional transformer is also quite accurate. This demonstrates that our technique is effective when applied to medical symptom recognition in Chinese.

In this study, the model's F-score in 8 labels is greater than 90%, demonstrating that our model performs well in terms of classification accuracy and breadth (Additional file 1: Table S3). It is noted that recall can the thorough of a model, while precision show its accurate. The F-key score's tenet is that, while attempting to increase precision and recall, we also seek to reduce the discrepancies between them so that the F-score can more accurately assess the benefits and drawbacks of the model.

**Fig. 3:**
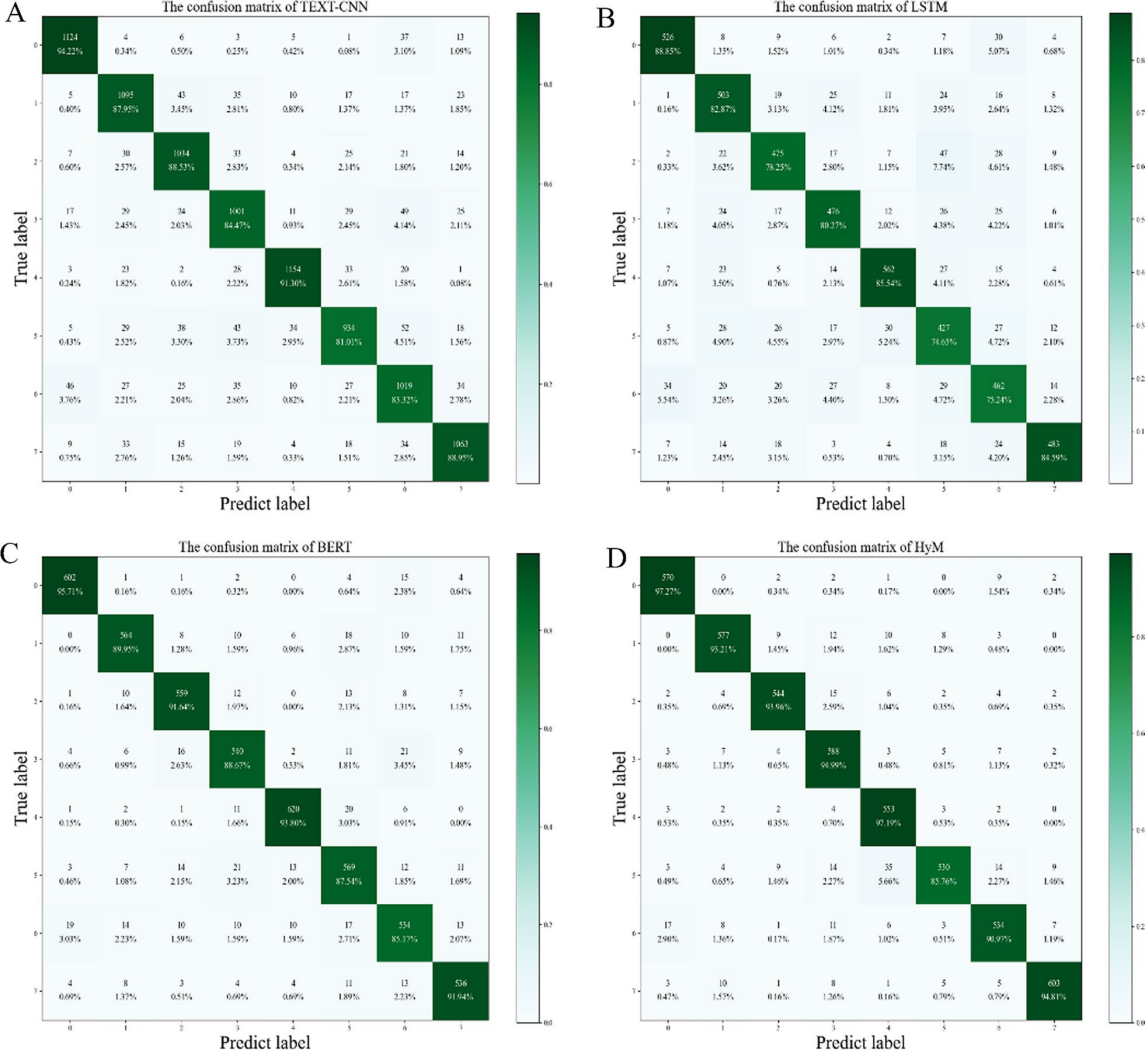
The confusion matrices (8 × 8) of four different model. **A** The confusion matrix of TEXT-CNN. **B** The confusion matrix of LSTM. **C** The confusion matrix of BERT. **D** The confusion matrix of HyM. The row indicates the number of data instances belonging to this class, and column of the confusion matrix indicates the number of data instances that has been predicted as this category. The confusion matrix is used to summarize the results of a classifier, where the closer the color is to the dark green in the middle of the picture, the more accurate the model is. From the four aboved pictures, the confusion matrix show that the HyM is superior to traditional classification models (TEXT-CNN, LSTM, BERT). TEXT-CNN, text convolutional neural networks; LSTM, long short-term memory; BERT, bidirectional encoder representations from transformers; HyM, Hybrid Model

The confusion matrices for each model are shown in Fig. 3. TEXT-CNN performs well in the classification of texts with labels 0 and 4. However, the classification outcomes for other labels are broad. The findings of label 5 and label 6 are only 74.65% and 75.24%, respectively, and the overall classification effect of LSTM is not as excellent as that of TEXT-CNN. For BERT, labels 0, 2, 4, and 7 all received above 90% of the results. Moreover, label 5 has the best result (87.54%). It is clear that our model HyM performs well across the board and in some test areas even achieves 97.3% accuracy.

The proposed model performs well in multi-class text classification when compared to other models. In conclusion, this study's evaluation indicators for the model are superior to those for other classification models, showing that HyM can significantly enhance the accuracy of patient symptom classification.

## Conclusion

In this study, we combine the patient's symptoms, local and contextual semantic aspects of the text, and an attention mechanism to acquire global sentence features, which can determine the relevance of individual words. Experimental results show that the proposed model outperforms LSTM, TEXT-CNN, and BERT in terms of classification precision and performance stability. The classification model achieves greater than 93.5% accuracy and has a high capacity for generalization, outperforming conventional classification models. In the Chinese Disease Classification study, Yuehjen Shao [31] proposed a new hybrid intelligent modeling scheme to obtain different sets of explanatory variables, and the proposed hybrid models effectively classify heart disease, However, this method is dependent on clinical indicators and cannot be classified. Chunhua Ju proposed online prediagnosis doctor recommendation model would improve patients’ online consultation experience and offline treatment convenience [32]. But this system's accuracy in studies to categorize five medical specialties was always lower than 70%, making it unusable in real-world settings. But our study shows the ability to automate the classification of patients’ questions more accurately and in real-time, which is very important implication in clinics. It will help to save the resources and time, as well as, lessens doctors’ and practitioners’ efforts in directing the patients to correct specialties.

This research can be expanded to research niches in more depth. As proposed in this study, HyM can be used for network interrogation and triage, intelligent hospital guidance, and medical text data mining as well as processing. In the future, we intend to develop a more colloquial dataset to match the patient's symptoms, and to investigate the role of attention mechanism technique and hyperparameter settings. In addition, we will consider incorporating additional elements such as disease symptom texts, medical numerical data, medical text dictionaries, and other characteristics in order to construct a more comprehensive and practical categorization model.

## Supplementary Information

**Additional file 1: Fig. S1.** The TF-IDF module of the HyM model, which is used to record the uniqueness and important position of the word in the document. **Fig. S2.** The TEXT-CNN module of HyM model for remembering more contextual information. **Fig. S3.** The LSTM module of the HyM model represents the deep level features of disease symptom text as vectors. **Fig. S4.** The BERT module of HyM model, the attention process prioritizes important content while ignoring less important content. **Table S1.** Sample instance of the Chinese patient description text dataset. **Table S2.** The accuracy of each model. **Table S3.** Experimental Results of each model.

## Data Availability

The online data can be accessed through the website located at www.39.net. The offline data can be accessed using the website located at www.xywy.com. All of the raw data are analyzed in this paper. The datasets used and/or analyzed during the current study are available from the author (maoch3@foxmail.com) on reasonable request.
