# Supplementary material for: Automatic medical specialty classification based on patients’ description of their symptoms
Source: BMC Med Inform Decis Mak. 2023 Jan 20;23:15. doi: 10.1186/s12911-023-02105-7 (PMC9862953; doi:10.1186/s12911-023-02105-7)
Supplement: Supplementary file 1 — Additional file 1: Fig. S1. The TF-IDF module of the HyM model, which is used to record the uniqueness and important position of the word in the document. Fig. S2. The TEXT-CNN module of HyM model for remembering more contextual information. Fig. S3. The LSTM module of the HyM model represents the deep level features of disease symptom text as vectors. Fig. S4. The BERT module of HyM model, the attention process prioritizes important content while ignoring less important content. Table S1. Sample instance of the Chinese patient description text dataset. Table S2. The accuracy of each model. Table S3. Experimental Results of each model. [file 12911_2023_2105_MOESM1_ESM.docx]

**Supplementary Information**

**Automatic Medical Specialty Classification Based on Patients’ Description of their Symptoms**

Chao Mao^1^**^﹟^**, Quanjing Zhu^2^**^﹟^**, Rong Chen^3^, Weifeng Su^1^*****

*1* *Guangdong Provincial Key Laboratory of Interdisciplinary Research and Application for Data Science, BNU-HKBU United International College, Zhuhai 519087, China*

*2* *Specialty of Laboratory Medicine, West China Hospital, Sichuan University,* *Guoxue Lane, Wuhou District, Chengdu 610041, China*

*3* *Specialty of Rehabilitation Medicine, The First Affiliated Hospital, Sun Yat-sen University, Guangzhou 510080, China*

***** Corresponding author

E-mail addresses: [wfsu@uic.edu.cn](mailto:wfsu@uic.edu.cn)

**﹟**: Equal contribution by the first two authors

[Fig S1. The TF-IDF module of the HyM model, which is used to record the uniqueness and important position of the word in the document 3](#_Toc124941228)

[Fig S2. The TEXT-CNN module of HyM model for remembering more contextual information 4](#_Toc124941229)

[Fig S3. The LSTM module of the HyM model represents the deep level features of disease symptom text as vectors 5](#_Toc124941230)

[Fig S4. The BERT module of HyM model,the attention process prioritizes important content while ignoring less important content. 6](#_Toc124941231)

[Table S1. Sample instance of the Chinese patient description text dataset 7](#_Toc124941232)

[Medical specialty 7](#_Toc124941233)

[Table S2. The accuracy of each model 8](#_Toc124941234)

[Table S3. Experimental Results of each model 9](#_Toc124941235)


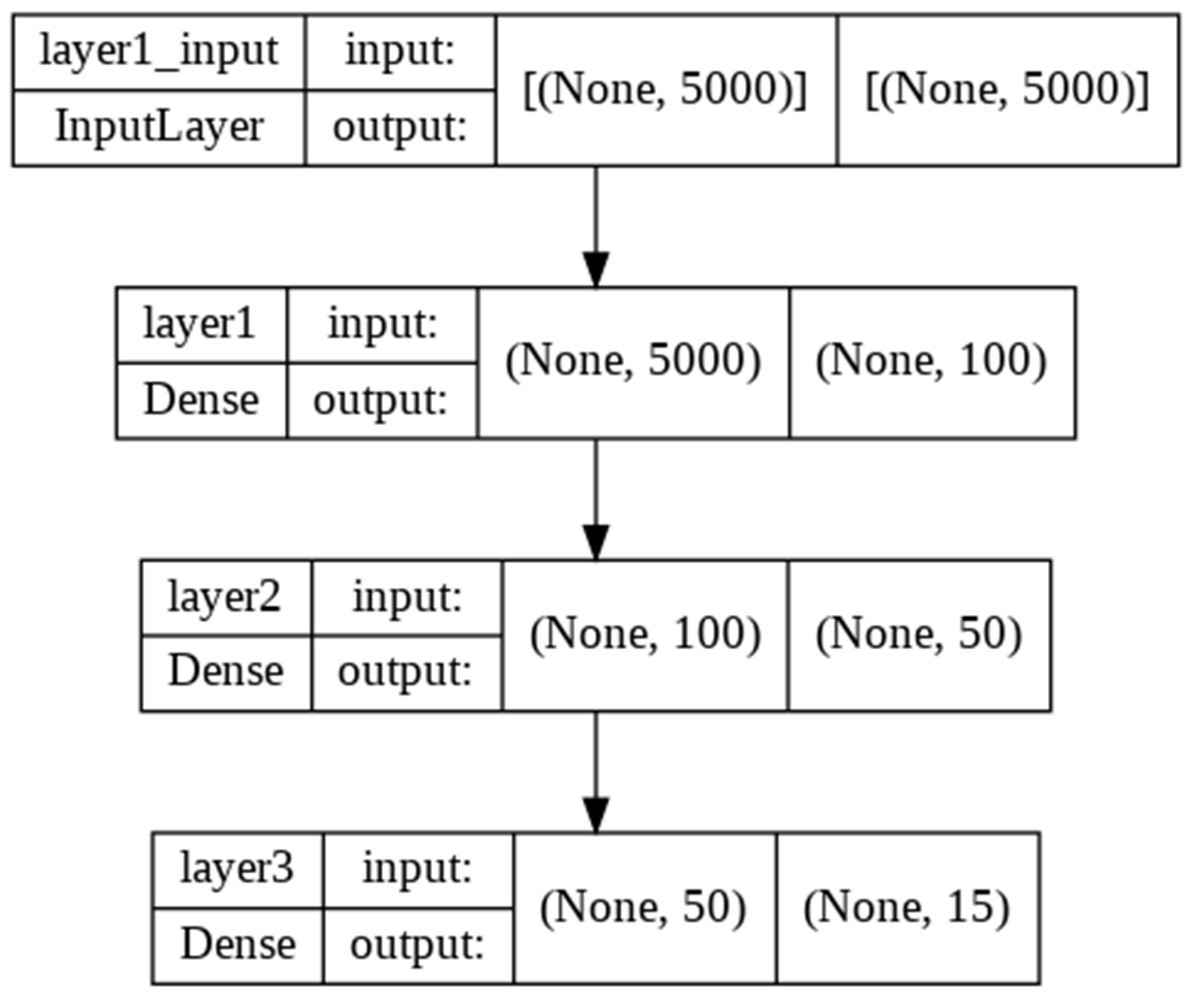


# Fig S1. The TF-IDF module of the HyM model, which is used to record the uniqueness and important position of the word in the document


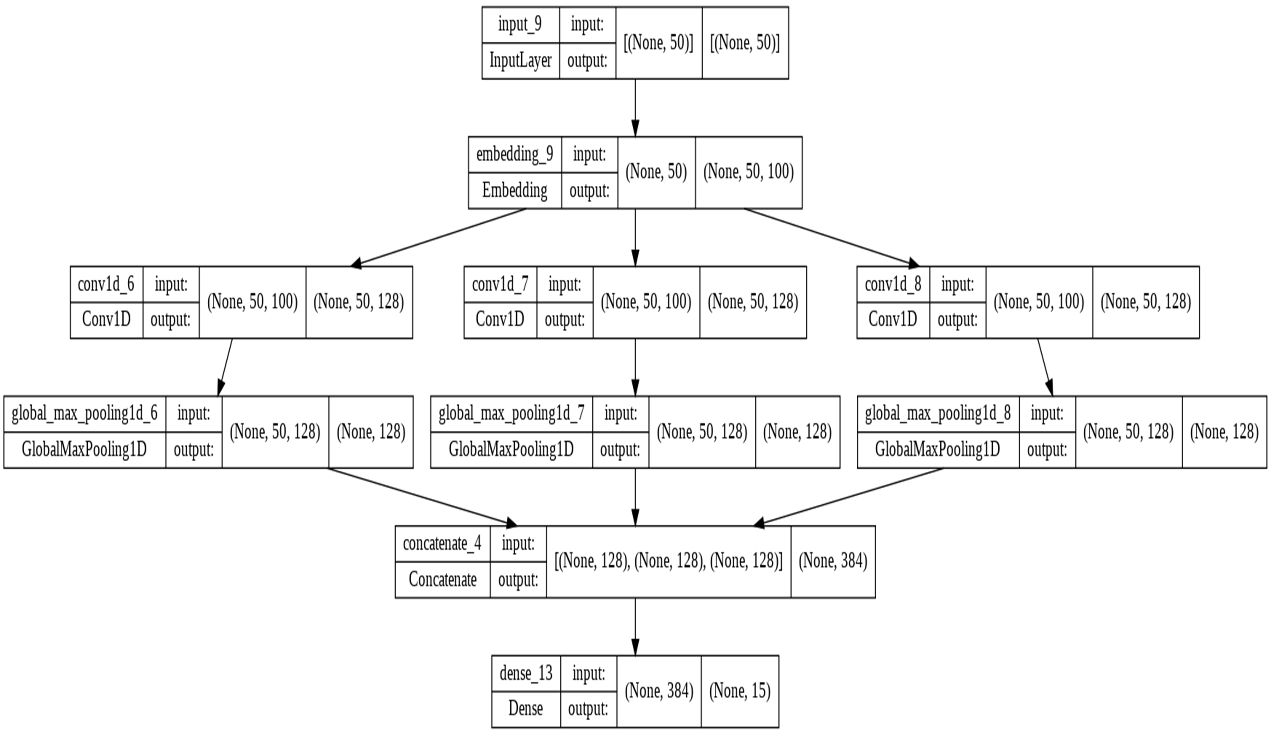


# Fig S2. The TEXT-CNN module of HyM model for remembering more contextual information


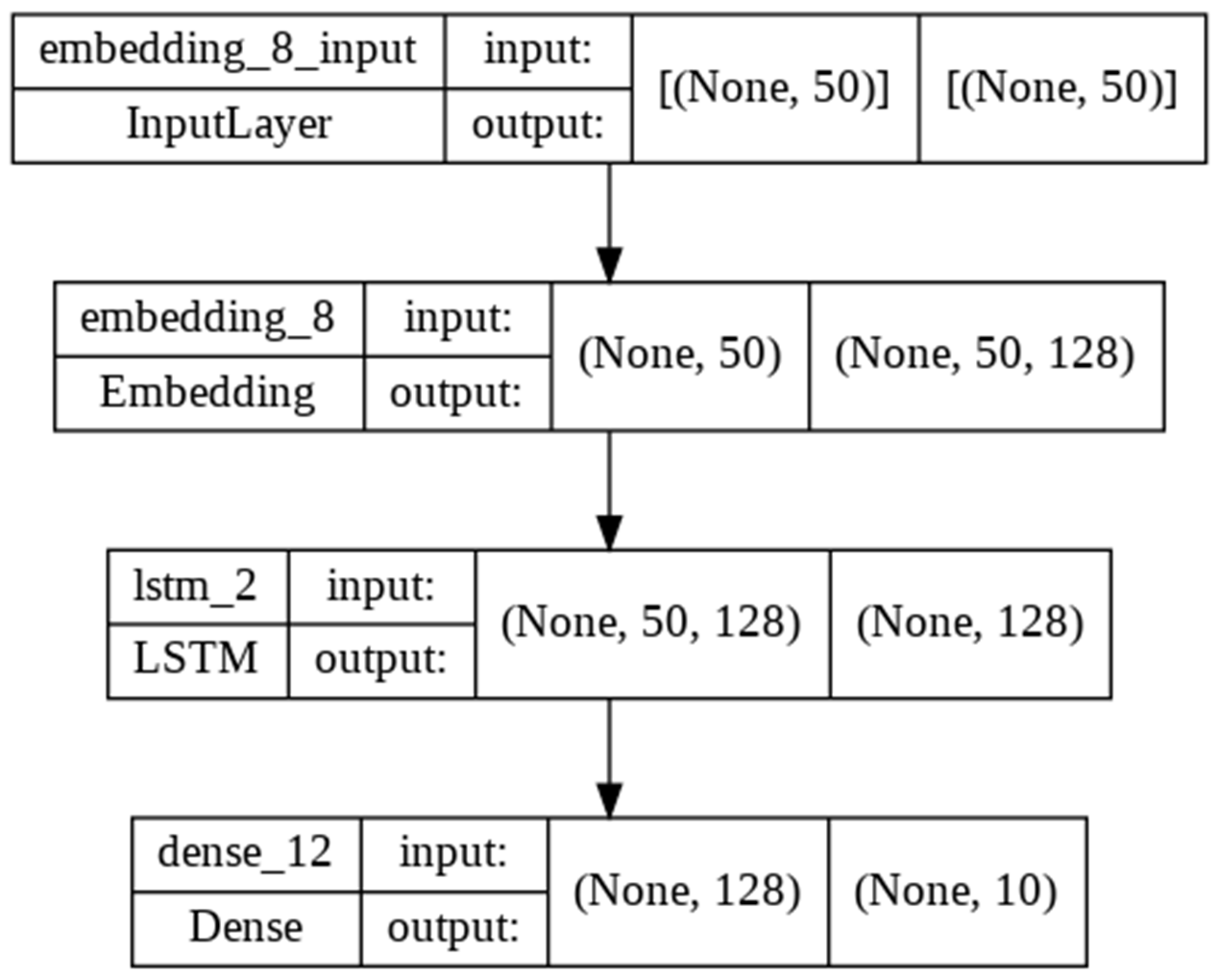


# Fig S3. The LSTM module of the HyM model represents the deep level features of disease symptom text as vectors


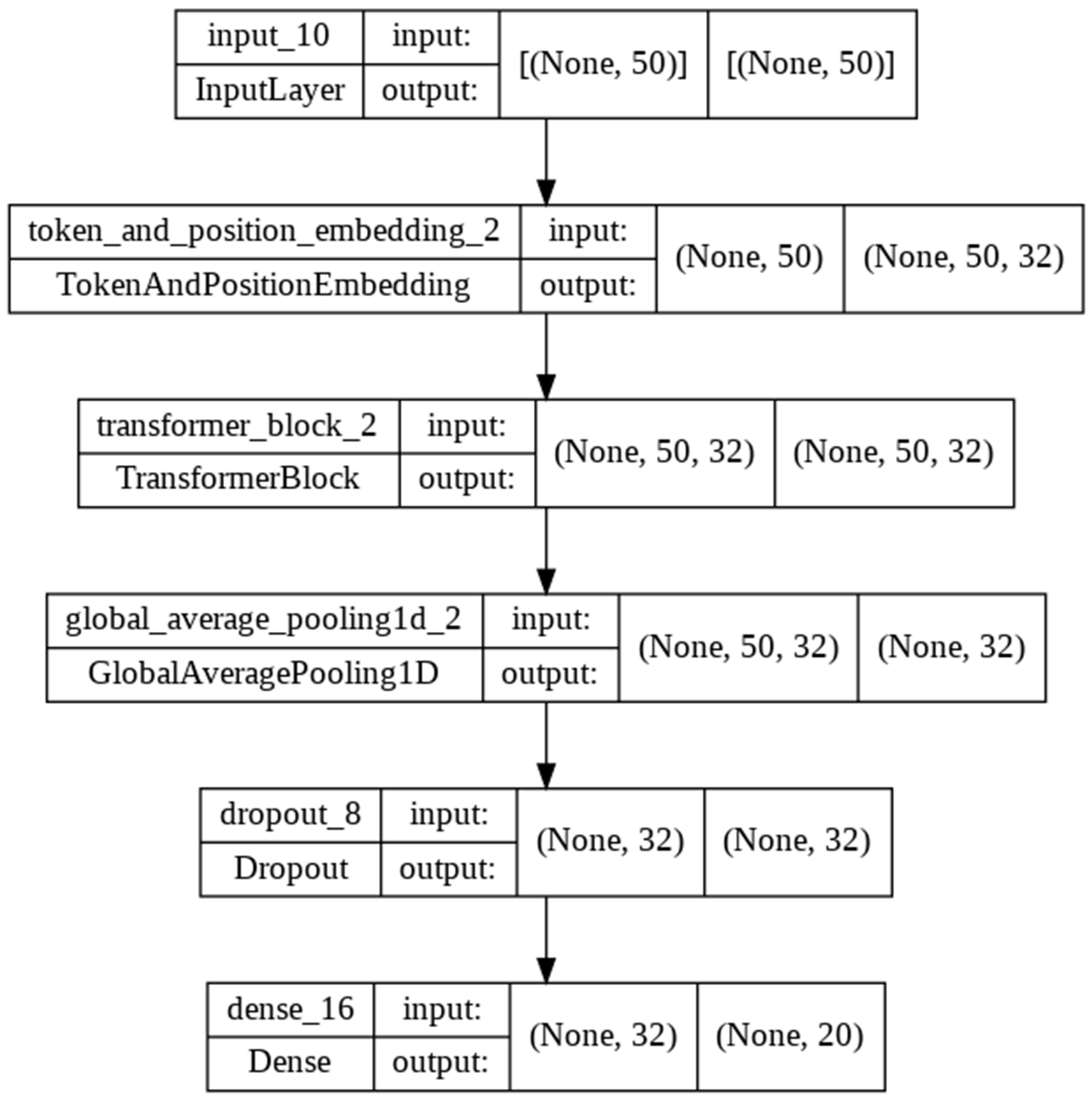


# Fig S4. The BERT module of HyM model,the attention process prioritizes important content while ignoring less important content.

# Table S1. Sample instance of the Chinese patient description text dataset

| **Patient's Symptoms** | Medical specialty |
| --- | --- |
| ^Does a man’s chest X-ray affect his pregnancy^ | ^Infertility^ |
| ^How to regulate internal heat in lung deficiency^ | ^Traditional Chinese Medicine^ |
| ^Why is the mouth crooked when speaking^ | ^Otorhinolaryngology^ |
| ^What is the matter with the child’s calf pain after a fever^ | ^Pediatrics^ |
| ^What is the matter with the heart sometimes^ | ^Internal Medicine^ |
| ^Can a calf fracture be operated on right away^ | ^Surgical department^ |
| ^Cesarean section eleven months pregnant^ | ^Obstetrics and Gynecology^ |
| ^What are the symptoms and dangers of prostatitis^ | ^Andrology^ |

# Table S2. The accuracy of each model

| **Models** | **Accuracy** |
| --- | --- |
| TEXT–CNN | ^0.887^ |
| LSTM | ^0.813^ |
| Bert | ^0.905^ |
| HyM | ^0.935^ |

# Table S3. Experimental Results of each model

| **Model** | **Specialty** | **Precision** | **Recall** | **F-score** |
| --- | --- | --- | --- | --- |
| ^TEXT-CNN^ | ^Infertility^ | ^0.924^ | ^0.941^ | ^0.932^ |
|  | ^Traditional Chinese Medicine^ | ^0.77^ | ^0.906^ | ^0.833^ |
|  | ^Otorhinolaryngology^ | ^0.888^ | ^0.85^ | ^0.869^ |
|  | ^Pediatrics^ | ^0.875^ | ^0.841^ | ^0.858^ |
|  | ^Internal Medicine^ | ^0.919^ | ^0.927^ | ^0.923^ |
|  | ^Surgical department^ | ^0.775^ | ^0.86^ | ^0.815^ |
|  | ^Obstetrics and Gynecology^ | ^0.879^ | ^0.754^ | ^0.812^ |
|  | ^Andrology^ | ^0.94^ | ^0.856^ | ^0.896^ |
| ^LSTM^ | ^Infertility^ | ^0.893^ | ^0.889^ | ^0.891^ |
|  | ^Traditional Chinese Medicine^ | ^0.783^ | ^0.829^ | ^0.805^ |
|  | ^Otorhinolaryngology^ | ^0.806^ | ^0.783^ | ^0.794^ |
|  | ^Pediatrics^ | ^0.814^ | ^0.803^ | ^0.808^ |
|  | ^Internal Medicine^ | ^0.884^ | ^0.855^ | ^0.869^ |
|  | ^Surgical department^ | ^0.706^ | ^0.747^ | ^0.726^ |
|  | ^Obstetrics and Gynecology^ | ^0.737^ | ^0.752^ | ^0.745^ |
|  | ^Andrology^ | ^0.894^ | ^0.846^ | ^0.869^ |
| ^Bert^ | ^Infertility^ | ^0.9495^ | ^0.9571^ | ^0.9533^ |
|  | ^Traditional Chinese Medicine^ | ^0.9216^ | ^0.8995^ | ^0.9104^ |
|  | ^Otorhinolaryngology^ | ^0.9134^ | ^0.9164^ | ^0.9149^ |
|  | ^Pediatrics^ | ^0.8795^ | ^0.8867^ | ^0.8831^ |
|  | ^Internal Medicine^ | ^0.9466^ | ^0.938^ | ^0.9422^ |
|  | ^Surgical department^ | ^0.8582^ | ^0.8754^ | ^0.8667^ |
|  | ^Obstetrics and Gynecology^ | ^0.8627^ | ^0.8517^ | ^0.8571^ |
|  | ^Andrology^ | ^0.9069^ | ^0.9131^ | ^0.91^ |
| ^HyM^ | ^Infertility^ | ^0.948^ | ^0.973^ | ^0.960^ |
|  | ^Traditional Chinese Medicine^ | ^0.943^ | ^0.932^ | ^0.937^ |
|  | ^Otorhinolaryngology^ | ^0.951^ | ^0.940^ | ^0.945^ |
|  | ^Pediatrics^ | ^0.899^ | ^0.950^ | ^0.924^ |
|  | ^Internal Medicine^ | ^0.899^ | ^0.972^ | ^0.934^ |
|  | ^Surgical department^ | ^0.923^ | ^0.858^ | ^0.903^ |
|  | ^Obstetrics and Gynecology^ | ^0.924^ | ^0.910^ | ^0.917^ |
|  | ^Andrology^ | ^0.965^ | ^0.948^ | ^0.956^ |
